# Using Optimal Control to Disambiguate the Effect of Depression on Sensorimotor, Motivational and Goal-Setting Functions

**DOI:** 10.1371/journal.pone.0167960

**Published:** 2016-12-14

**Authors:** He Huang, Katia Harlé, Javier Movellan, Martin Paulus

**Affiliations:** 1 Department of Cognitive Science, UC San Diego, La Jolla, California; 2 Machine Perception Lab, UC San Diego, La Jolla, California; 3 Laureate Institute for Brain Research, Tulsa, Oklahoma; 4 Department of Psychiatry, UC San Diego, La Jolla, California; Universita degli Studi di Napoli Federico II, ITALY

## Abstract

Differentiating the ability from the motivation to act is of central importance to psychiatric disorders in general and depression in particular. However, it has been difficult to develop quantitative approaches to relate depression to poor motor performance in goal-directed tasks. Here, we use an inverse optimal control approach to provide a computational framework that can be used to infer and factorize performance deficits into three components: sensorimotor speed, goal setting and motivation. Using a novel computer-simulated driving experiment, we found that (1) severity of depression is associated with both altered sensorimotor speed and motivational function; (2) moderately to severely depressed individuals show an increased distance from the stop sign indicating aversive learning affecting goal setting functions. Taken together, the inverse optimal control framework can disambiguate on an individual basis the sensorimotor from the motivational dysfunctions in depression, which may help to develop more precisely targeted interventions.

## Introduction

*Sensorimotor skills*, *motivation*, and *goal setting* can all affect performance in complex ways. It is important to distinguish their effects to understand observed individual differences in any goal-directed motor task. At present, most of the experimental paradigms are restricted to observing the behavior of discrete actions and using reaction time as the measure for performance. However, it is difficult to distinguish those factors from discrete actions or reaction time, because those factors jointly influence both the cognitive control (movement planning) and movement execution [1]. For example, in a goal-directed motor task, a slower action may be caused by slower movement execution due to impaired sensorimotor system; or by different goals, for instance, minimizing the control noise with slower velocity; or lack of motivation, i.e., not willing to spend effort to achieve the goal. Thus it is difficult to investigate their individual influence from the confounded result.

Dissociating how these three factors conspire to explain observed behavior has profound implications for the treatment of mood disorders [2], such as major depressive disorder (MDD). Mood [2] and anxiety [3] disorders will account for approximately $16 trillion lost productivity or 25% of global GDP over the next 20 years [4] and are among the most common and devastating mental health conditions worldwide. The clinical presentation of depression can be remarkably varied, with some MDD patients exhibiting anhedonia, sleeplessness, excessive guilt, and psychomotor slowing, while other MDD patients exhibit hypersomnia, heightened interpersonal sensitivity, and psychomotor agitation. There is no evidence that antidepressants have direct motor effects [5, 6]. There is a possibility that SSRI or other antidepressants indirectly affect motor behavior via their changes in mood, motivation, or executive functioning, although the evidence is weak [7]. On the other hand, there is strong evidence that anti-anxiety medications, e.g. benzodiazepines, have significant effects on motor behavior in general and driving behavior in particular [6,7]. Thus, one would need to assess these effects in depressed individuals who are receiving these medications. Unfortunately, traditional diagnostic methods, like self-reports, clinician ratings, and performance in simple tasks, were not designed to disambiguate the effect of these three factors. It is therefore not surprising that conflicting evidence has been found regarding the extent to which depression is associated with sensorimotor, motivation, and/or goal-setting factors [8]. Thus, a precise delineation of these deficits can help to develop more targeted interventions in the future.

We previously compared the performance of healthy controls and depressed individuals in a simulated driving task [9], where subjects were instructed to drive a virtual car to a stop sign as quickly as possible and stop as close as possible. In this task depressed individuals stopped further away from a stop sign. However, it may be interpreted in several possible ways. For example, this difference could be caused by slower speed to execute acceleration/deceleration actions, or by different performance criterions due to avoidance motivation, e.g., further intended stopping distance, or less effort one is willing to spend to achieve the intended stopping distance. Thus it is important to disentangle those factors to explain the causes of the observed behavioral difference. Here, we aim to further infer and isolate the cognitive processes underlying altered goal-directed behavior in depression, by developing an experimental paradigm that can independently assess sensorimotor speed, motivation, and goal setting functions, and applying a computational framework that can disambiguate their effect on observed behavior.

**Fig 1:**
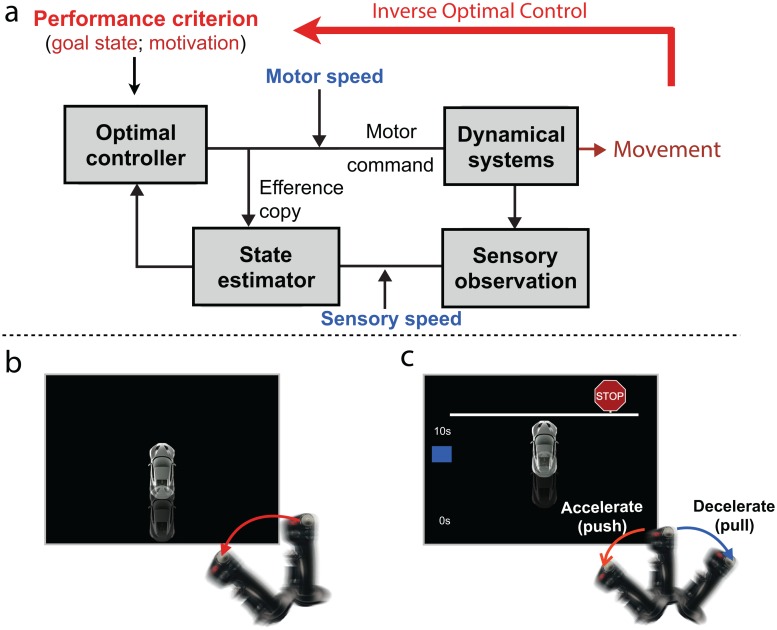
Computational framework and experiment design. a. Inverse optimal control framework. b. Task 1. c. Task 2.

Optimal control theory has been shown to be an effective computational framework to understand goal-oriented human behavior in a wide range of tasks, from eye-movements to complex object manipulation [10, 11]. This approach frames goal-oriented behavior as the solution to a constrained optimization problem in which a sensorimotor system (the human body) is used to maximize the achievement of a desired goal state while minimizing effort. The solution to this problem takes the form of a feedback controller that maps moment by moment, the history of sensory information into sequences of motor commands (Fig 1). In robotics this framework is typically used to develop control systems for robots to achieve predefined goals at minimum energy cost [12, 13]. The approach is also used in reverse engineering problems to infer the goals, costs, and algorithms that drive the behavior of a complex black-box system [14]. This is known as “inverse optimal control”. This inverse approach provides a mathematical model of how sensorimotor, motivational and goal-setting influences interact with each other to produce goal-oriented behavior.

To independently assess sensorimotor, goal-setting, and motivation factors, we designed a two-task experiment based on the previous paradigm (Fig 1). In Task 1, the car would start to move from stationary state (at different speeds) and participants were instructed to push a joystick to an instructed position as soon and as quickly as possible once they observed motion of the car. In Task 2, similar as in previous study, subjects were instructed to drive the car as quickly and as close as possible to a stop sign. Task 1 is aimed to measure individual’s sensorimotor speed parameters, and Task 2 is aimed to measure motivation and goal setting parameters. We hypothesize that depressive behavior maybe reflected by changes in sensorimotor, motivation, and/or goal setting parameters.

## Materials and Methods

### Participants

66 college students (20 male and 46 female subjects, mean age (years) 20.6, std = 2.04, range 18–27) participated this study (approved by the Human Research Protections Program at University of California San Diego) in Fall quarter 2013 and Winter & Spring quarter 2014. They signed up through UCSD SONA system (an online experiment scheduling system used to recruit subjects in UCSD), completed phone-screening and on-line BDI (Beck Depression Inventory, BDI-II, [15]) measure. We aimed to recruit about 25% of participants with no significant depression level (i.e., BDI score<6) while the remaining part of recruited participants needed a BDI score >7. Other inclusion criteria included: being in good general health on the basis of brief review of medical history, and sufficient proficiency in English to understand and complete all study procedures. Exclusion criteria included: lifetime history of psychotic, bipolar or obsessive-compulsive disorder, history of current alcohol or substance dependence, recent history of (i.e., within last 6 months) or currently taking any antidepressant or psychotropic medications (except occasional sleep aid). Qualified subjects signed the informed consent, and completed the experiment (with a second BDI measured prior to the task) in the lab, and were compensated by 2 course credits. Their onsite BDI range from 0 to 39 with mean BDI = 12.59 (std = 10.55), median BDI = 10. For each parameter, we used both continuous BDI as the dependent variable and also examined depression groups based on Beck AT et al. 1996 [15] as follows: Non-dep (0≤BDI≤5, n = 17), Min-mild dep (6≤BDI≤19, n = 33), Mod-dep (20≤BDI≤28, n = 9), and Sev-dep (29≤BDI≤63, n = 7).

### Experiment

Subjects completed two tasks in this experiment. Both tasks were computerized tasks programmed using MATLAB (Mathworks) and the Psychophysics Toolbox on a 15 inch MacBook Pro. Subjects performed Task 1 twice (120 trials, before and after Task 2). In each trial, a car would appear on the bottom of the screen, and subjects were instructed to push the joystick from resting position forward to the maximum position as quickly as possible once they observe the car move. Each trial started with a 3-second countdown and a random waiting interval (1–3 seconds), then the car would start to move at a randomly selected speed (.01-.3 cm/second). Car speed range was picked to ensure an exponential decay of response time as the car speed increases. Trials ended once subjects pushed the joystick at its maximum forward position. For Task 2, subjects completed 60 trials split into 3 blocks of 20 trials each. In each trial, subjects were instructed to drive a virtual car as quickly as possible and stop at a stop sign (distance: 10.62 cm) without crossing the stop-line, with a fixed time window of 10-second. Each trial started with a 3-second countdown and ended when time ran out, with no performance feedback (e.g., points) in the end. Premature action on the joystick before the trial starts (i.e. holding the joystick in the maximum position) was considered as a false start and would abort and restart the current trial. They were given a 10 seconds practice to familiarize with the joystick control during instruction session. The car has a linear dynamic system, in which the car position is controlled by continuous joystick position. We recorded their continuous actions using a gaming joystick (Thrust-master HOTAS Warthog Flight Stick). The goal of Task 1 is to measure individual’s sensorimotor speed, and the goal of Task 2 is to apply inverse optimal control model to recover reward-function in a goal-directed task (see Fig 1).

### Models

An inverse optimal control model was used to distinguish sensorimotor speed, goal setting and motivational effects in observed behavior. To achieve that, we first assessed individual’s sensorimotor system by estimating their sensory speed and motor speed in Task 1. Then we estimated their goal state (intended stopping distance) and motivation (the amount effort one is willing to spend to achieve the goal state) in the reward function in Task 2, taking account of the sensory and motor speed parameters from Task 1. Model simulation for the effects of the three parameters of interest is shown in Figure A in S1 Fig.

#### Sensory-motor speed

Task 1 was designed to estimate sensory speed γ and motor speed β. Sensory speed measures the delay between the actual car position and the observed car position. Faster sensory speed will improve how fast sensory information was used in estimating the car’s state (e.g. position, velocity). Motor speed measures the lag between the desired action and the actual action. Faster motor speed will improve how quickly motor command on the joystick was carried out.

We model subjects’ perceived car position *Yt* as a delayed true car position *Xt* due to the limit of sensory processing speed γ. The higher the γ, the closer the perceived car position *Yt* is to the true car position *Xt*. We assume subjects will decide the car starts moving once the perceived car position *Yt* reaches a position threshold *Xthd*. Thus the minimal time for the perceived car position *Yt* to reach the threshold *Xthd* is reaction time *RT*:

Perceived car position *Yt*:

$$dY_{t}=\;\gamma \left(X_{t}-Y_{t}\right)dt$$ (1)

Reaction Time:

$$RT=\;argmin_{t}\left\{Y_{t}\geq X_{thd}\right\}$$ (2)

We model joystick position *Ct* as a delayed execution from target joystick position *U*_*target*_, due to the limit of motor execution speed β. The higher the β, the closer joystick action is to the desired target position. Thus the minimal time for *Ct* to reach *Utarget* is movement time:

Joystick position *Ct*:

$$dC_{t}=\;\beta \left(U_{target}-C_{t}\right)dt$$ (3)

Movement Time:

$$MT=\;argmin_{t}\left\{C_{t}\geq U_{target}\right\}$$ (4)

In above equations, *Xt* (true car position), *RT* (reaction time to car motion-onset), *Ct* (recorded joystick position), *Utarget* (target position) and *MT* (movement time) are known. Reaction time to car motion-set and true car position were used to recover γ, and recorded joystick action and movement time were used to recover β, by applying Maximum Likelihood Estimation (i.e. optimizing over γ, β over predicted reaction time, movement time and observed data.).

#### Goal state and motivation

Task 2 was designed to estimate individual’s reward-function. It is a function of goal stopping distance (*goal state*) and the ratio between internal reward for achieving the goal and the energy expenditure (*motivation*). *Goal state* measures individual’s intended stopping distance from the stop sign. *Motivation* measures individual’s willingness to reach the goal stopping distance. In a quadratic reward function, *goal* represents the optimal point of the reward function, and *motivation* represents the hessian of the reward function. We formulate the driving task as a Linear Quadratic Gaussian (LQG) problem[16] with a linear dynamic system taking into account of the sensorimotor speed estimated from Task 1, and a quadratic reward function of *goal* and *motivation*.

Assuming the driving task as a linear dynamic system Eq (1) with a partial hidden state *Xt* and observable feedback *Zt*, in which *Xt* is a vector including the (hidden) true car distance to target stopping position at time *t*, joystick action at time *t*, and perceived car distance to target stopping position at time *t*.

Linear dynamic system:

$$dX_{t}=AX_{t}dt+BU_{t}dt$$ (5)

Observation:

$$Z_{t}=CX_{t}+V_{t}$$ (6)

In which, A is a dynamics matrix with motor and perceptual speed estimated from Task 1 and parameters of car dynamics (assuming known), B is input matrix which takes into consideration of subject’s motor speed, *Ut* is the optimal internal action command, and *Vt* is Gaussian noise. Model details are provided in S1 appendix.

We assume the reward function *r* (*Xt*, *Ut*) is a quadratic function of current state *Xt* and action *Ut* Eq (7). It evaluates current state *Xt* based on its distance from the goal state (*G)* and the ratio of the weight on this distance over the energy expenditure, which is defined as motivation *M* in our framework.

Reward function:

$$r\left(X_{t},U_{t}\right)=g\left(X_{t},G,M\right)-U_{t}^{2}$$ (7)

### Statistical analysis approach

To examine the influence of depression severity on sensorimotor speed, goal setting and motivation, for each of the parameter, we first performed linear regression using BDI as the dependent measure, and then conducted ANOVA for depressive groups analysis. For goal setting and motivation parameter, we used linear mixed effect models [17] with subject modeled as a random effect, and examined the main effect of depression using BDI as fixed effect, and the interaction between BDI and block using BDI and block as fixed effects.

## Results

### Sensorimotor parameters

**Fig 2:**
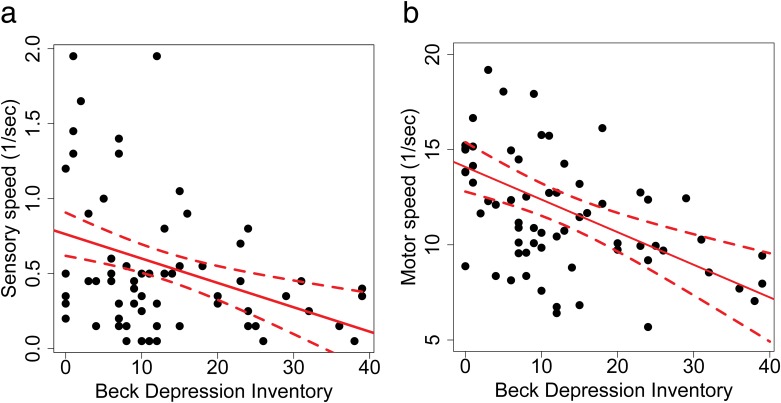
Sensorimotor speed. a. Sensory speed. b. Motor speed.

We estimated sensory speed from reaction time to car motion-onset (Eqs 1 and 2 in Material and Methods), and motor speed from recorded joystick position and time used to push the joystick to the target position (Eqs 3 and 4 in Material and Methods) in Task 1. Using BDI as the independent measure (Fig 2), both sensory speed and motor speed decrease significantly as depression severity increases (Sensory speed: *R*^2^ = .13, *F*(1,64) = 9.49, *p* = 9.49, *p* = .003; Motor speed: *R*^2^ = .17, *F*(1,64) = 13.1, *p* < .001). These results are consistent with a relatively longer reaction time to car motion-onset (*R*^2^ = .04, *F*(1,64) = 2.97, *p* = .08, n.s.) and a significantly longer movement time from resting to maximum forward position of the joystick (*R*^2^ = .18, *F*(1,64) = 13.8, *p* < .001). Separating by depressive groups, a one-way ANOVA among depressive groups also showed there was a significant main effect of depression on sensory speed (*F*(3,62) = 5.01, *p* = .004) and motor speed (*F*(3,62) = 6.4, *p* = .001). Group comparisons of sensorimotor speed are shown in Figure B in S1 Fig.

### Goal setting parameter

**Fig 3:**
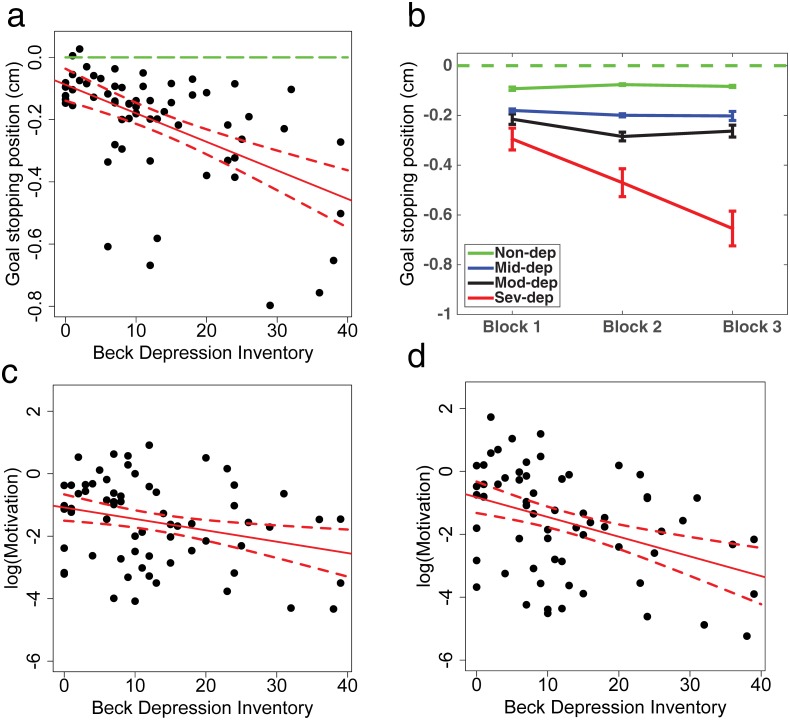
Goal stopping position and motivation estimated from Task 2. a. Average goal stopping position across blocks as a function of BDI. b. Goal stopping position over time in three blocks in four depressive groups. c. Estimated motivation for each subject, taking into consideration of individual differences in both sensorimotor speed and goal state. d. Estimated motivation for each subject, not considering individual differences in sensorimotor speed and goal state.

The goal-setting parameter here reflects the participants intended stop location, which was measured as distance from the stop sign. Negative values indicate that the participant intended to stop before the stop sign and positive values that he/she intended to stop after the stop sign. For each subject, we estimated their goal stopping position in each of the three experimental blocks. As shown in Fig 3a, individuals’ mean goal stopping position negatively correlates with BDI (*R*^2^ = .27, *F*(1,64) = 23.79, *p* < .001), which indicates as depression severity increases, subjects intended to stop further away from the stop sign. A one-way ANOVA among depressive groups also showed there was a significant main effect of depression on goal stopping position (*F*(3,62) = 10.91, *p* < .001, in Figure C in S1 Fig).

Using a linear mixed effect model, with subject modeled as a random effect and BDI as fixed effect, we found a significant negative effect of BDI (*B* = −.009, *p* < .001) on subjects’ goal stopping positions. Additionally, we also investigated how depression affected behavior over time in the three experimental blocks (Fig 3b). With subject modeled as a random effect, BDI and block as fixed effects in the linear mixed effect model, we found a significant interaction between BDI and block (*F*(2,132) = 8.43, *p* < .001) while no main effect of block was found (*F*(2,132) = .38, *p* > .1). Results of the full model are displayed in Table 1.

**Table 1:** Fixed effects for model predicting goal stop distance.

Parameter	Estimate	Pr (> |t|)
**Intercept**	-0.109659	0.00229 **
**BDI**	-0.005150	0.01801 *
**Block_2**	0.022257	0.46902
**Block_3**	0.043060	0.16239
**BDI: Block_2**	-0.004434	0.01926 *
**BDI: Block_3**	-0.007652	7.5e-05 ***

* < .5,

**: < .01,

***: < .001

More specifically, comparing to Block 1, Non-dep individuals had significantly closer goal distance in Block 2 (*B* = .02, *p* = .003), while Mod-dep individuals had significant further goal distance in Block 2 (*B* = −.07, *p* < .001). Sev-dep individuals had significant further goal distance both in Block 2 (*B* = −.18, *p* = .002) and Block 3 (*B* = −.36, *p* < .001). No significant change in Mid-dep group over blocks was observed (p > .1).

### Motivation parameter

Taking into account sensorimotor speed and goal state, motivation parameter for each individual subject was estimated in each of the three experimental blocks. Using BDI as the dependent measure, we found that mean motivation (log-transformed) has a mildly negative correlation with depressive severity (*R*^2^ = .06, *F*(1,64) = 4.31, *p* = .04, *B* = −.03, Fig 3c). A one-way ANOVA among depressive groups showed there was no significant main effect of depression group on motivation (*F*(3,62) = .69, *p* > .1). For comparison, we also examined how it would be reported differently if not considering individual differences in sensorimotor speed and goal state. In fact, in this case, the mean motivation (log-transformed) would have a stronger negative correlation with BDI (*R*^2^ = .16, *F*(1,64) = 11.76, *p* = .001, *B* = −.06, Fig 3d), and the one-way ANOVA would report a significant main effect of depression severity on motivation (*F*(3,62) = 3.3, *p* > .0.3). Next, using a linear mixed effect model, with subject modeled as a random effect, BDI and block as fixed effects, we found a significant block effect on motivation (*F*(2,132) = 6.91, *p* = .001, in Figure D in S1 Fig, while no significant interaction between BDI and block was found (*p* > .1).

Additionally, we examined the accumulative action (measured by accumulated joystick position in each trial) across blocks in each depressive group and found that non-dep (*p* = .05) and mid-dep (*p* = .03) groups had increased action over blocks. However, there was no significant change in action between Mod and Sev-dep groups. Based on the accumulative action cost generated from different reward functions, model simulation suggests optimal action cost increases as the goal stopping distance decreases (closer to stop-sign) and as motivation increases (willing to spend more effort to achieve goal stopping distance). Taking into account each individual reward function with previously estimated goal state and motivation, we can map their optimal action cost estimated from the model. Result shows depressed individuals used the reward function that associated with the lowest action cost and is significantly lower from non-dep group (*T*(23) = −2.8799, *p* = .0085). Model simulation and mapped action cost is shown in Figure E in S1 Fig). A summary of correlation among model parameters and BDI (pairwise mutual information [18] and correlation coefficient) is provided in Figure F in S1 Fig.

## Discussion

We used an inverse optimal control modeling approach to parse the observed simulated driving behavior of individuals with a range of depressive symptoms, into three independent components: sensorimotor speed, goal-setting, motivation. We found that, relative to healthy controls, individuals with depressive symptom severity showed (a) slower sensorimotor speed and attenuated motivation, (b) increasing goal distance from the instructed target. This study is based on the notion that computational psychiatry approaches [19] aimed at arriving at a computational account of how psychiatric disorders impair neural and cognitive dysfunction [20] can be useful to disambiguate complex behavioral syndromes. In particular, the current results show that motivational components can be clearly delineated from psychomotor speed, which is important for the interpretation of psychiatric deficits.

### Sensorimotor parameters

Our results are consistent with prior work suggesting that depressed individuals have impaired sensorimotor pathways [8, 21], and suggest a tangible impact on complex goal-directed actions such as driving, independently of motivational factors. In fact, psychomotor retardation is one of the core symptoms in Major Depressive Disorder [22]. For example, relative to healthy controls, depressed individuals exhibit increased reaction times [1] and lower velocity [23] (for a comprehensive review, see [24]). However, traditional experimental methods often fail to identify whether slowing is from pure motor factors and/or from effort-based motivational factors, since motor slowing affects both motor and cognitive processes [1, 25]. Thus distinguishing the ability to act (motor slowing) from the motivation to act (motivation deficits) is critical in examining the impact of depression on performance in goal-directed motor tasks. The experimental paradigm proposed in this study provides one solution to this problem, by assessing independently the motor component from the (effort-based) motivation in a dual-task design. Since goal setting and motivation influence any human action, we cannot completely rule out that the behavior observed in Task1 was influenced by individual’s internal reward function. However, given explicit task instruction and the goal state (push the joystick to the maximum forward position once you see the car starts moving) in the absence of explicit positive or negative valence outcomes, we assume the behavior reflects the individuals’ baseline sensorimotor ability with a minimal influence of subjective reward function. Although the observed sensorimotor speed may be influenced by individual’s motivation, using the baseline sensorimotor ability as a covariate provides a within-subject control for Task 2 (i.e. relative to Task1). It is also worth noting that without controlling for the motor factor, we would have concluded very different result of the motivation parameter with the ANOVA analysis (e.g., Fig 3d). However, in the future it will be interesting to explore how individual’s motor speed may be affected by motivation in different context. For example, with reward or punishment associated with movement time. Our results provide support for helping depressed patients with impaired sensorimotor function, for instance by encouraging increased physical activity, which could be considered as one possible targeted intervention to improve patients with slower sensorimotor speed. Structured exercise is indeed one of the non-pharmacological interventions, which has proven helpful for depressed mood [26].

### Goal setting parameters

We found that, as depression severity increased, participants set goals further and further away from the stopping sign (and closer to self). Note that with no significant change in accumulated action across blocks, this different goal setting in the Sev-dep group is more likely to indicate a higher avoidance motivation rather than increased fatigue. This finding supports earlier work by Ahrens et al. [27] that depressed subjects (BDI> = 9) set lower personal goals (including work, home and social contexts) relative to non-depressed individuals, although there has been mixed findings in that area [28]. It is important to note, however, that depression is also associated with a lower tolerance and higher avoidance of negative outcomes [29], which could explain the observed gradation in distance from target over time among depressed individuals. For example, depressive individuals learn faster to avoid risky gambles [30] and demonstrate faster motor response to withdraw from negative stimuli such as negative faces [31]. Studies have also shown that depression is associated with more avoidant schemas and emotions [32]. For example, in a history-dependent decision-making task, Maddox et al. 2012 [33] showed that depression enhances loss-minimization, but not gain-maximization. In particular, depressed individuals may have a conditionally set goal [34], i.e. framing the task in terms of avoidance (to not cross the stop sign), as opposed to approach (e.g. to stop as close as possible to the stop sign). Thus it is possible that in tasks with potential punishment (e.g. crossing the stop sign), depressed individuals set a closer position goal to avoid ‘failure’ of crossing the line and minimize loss in the task. In addition, it will lead to higher likelihood of positive self-reward, which can be critical to maintain behavior without external rewards [27]. Furthermore, this less specific goal setting (i.e. stop further away from the ‘stop-sign’ vs. stop at the ‘stop-sign) is also consistent with recent report by Dickson et al. [35], in which they found that depressed individuals had reduced specificity of personal goals.

### Motivation parameters

Conceptually motivation may be related to ‘arrival time’ of individual’s goal stopping position. However in our computational framework, motivation was not derived from the arrival time, but was estimated through a quadratic reward function that is based on inverse LQG framework (Linear-Quadratic-Gaussian), using the continuous action and states recorded in participants’ data. Controlling the effects of individuals’ sensorimotor speed and goal-state, we found that in the stop-sign task, there is only mildly negative correlation between BDI and motivation. In comparison, a stronger negative correlation between BDI and motivation would be reported if individuals’ sensorimotor speed and goal-state were not controlled. This suggests that in order to investigate task-specific motivation in a goal-directed motor task, it is important to isolate the motor effect independently of the task and also consider individuals’ subjective goal state in the task. However, one possibility is that one factor may still influence the other, for instance depressed individuals may draw improper conclusion of the degree of lack of motivation from their slower sensorimotor speed and/or different goal-state. The results further indicate that motivation deficits may only be present in more severely depressed individuals, while for individuals with minimal to moderately depression, their behavioral difference are mainly in sensorimotor slowness and subjective goal setting. Thus, for those non-severely depressed, the conceptualization of depression as anhedonia and lack of motivation could be actually secondary to the primary effects depression on the motor system.

Anhedonia is a core symptom of depression and recent work has shown a significant inverse relationship between anhedonia and the willingness to expend effort to achieve goals [36]. As shown in our model, both further distance goal to the stop-sign and lower motivation will lead to lower action costs in the task. This suggests that for minimally to moderately depressed individuals (i.e., BDI < 29), the lower action cost is caused by setting a more distant stopping position goal, while for severely depressed individuals (BDI>29), the lower action cost is a combination of further stopping distance and lower motivation. Thus our findings provide important evidence of the difference in how anhedonia affects different aspects of an individual’s reward-function, which can be used to design more effective treatment plans for anhedonic depressed patients. In addition, to help patients with motivation deficits, finding proper reinforcement that can encourage subjects to spend more effort to achieve their goals may be a more promising treatment direction to investigate. Clery-Melin et al. [37] showed that depressed patients exerted more effort following emotionally arousing pictures, but not for higher monetary incentives. In future work, we plan to test the usefulness of various types of reinforcement using our task and to quantify their effects using the inverse optimal control model.

### Limitation

One limitation of the current study is not considering motor learning effect, as it may covary with goal setting and motivation. For future work, we will take into consideration of motor learning effect in the model, for example, by including motor noise in the model. Another limitation of current study includes the use of a self-report clinical measure to assess depression severity (BDI) and absence of psychiatric diagnostic classification in our sample. While this makes the relevance and generalization of our results difficult for clinically depressed individuals at more advanced stages of the disease, this approach emphasizes ecological validity (i.e., with a range of symptoms in the anxiety/neurotic and anhedonic affective dimensions) and may be particularly useful for early detection efforts of depressive symptomatic types within young adults at risk for developing full-blown clinical depression. In addition, examining the current definition of Major Depressive Episode, Watson reported [38] that although more than half of the DSM symptom criteria are so strongly correlated as to be nearly interchangeable, the remaining symptoms (e.g., insomnia, appetite loss) are only weakly to moderately interrelated. In that regard, he found that IDAS, Beck Depression Inventory, and possibly other depression scales are virtually indistinguishable. Thus, taken together, although individual depression measures are good indicators of general negative mood, they are not well suited to parse particular components that are specific to depression or are able to subtype depression.

## Conclusions

Our findings provide the first model-based evidence of distinct cognitive alterations among depressed individuals in sensorimotor speed, goal-setting, and motivation during a complex goal-directed motor task, confirming that depression is a cognitively intricate and multifold illness. Our results suggest that different treatment plans may be identified and emphasized to target individuals with different types of depressive symptoms, for example, physical training for individuals with sensorimotor deficits, and positive reinforcement training for individuals with poor goal-settings and lower motivations. Importantly, our modeling approach and motor-control paradigm offer a much needed computational scaffold for investigating, inferring, and more thoroughly understanding the neural basis of depression and associated cognitive deficits.

## Supporting Information

S1 Appendix Inverse Linear Quadratic Gaussian Model (LQG). (DOCX)

S1 Fig Supplementary modeling result figures. (DOCX)
