# Supplementary material for: Using Optimal Control to Disambiguate the Effect of Depression on Sensorimotor, Motivational and Goal-Setting Functions
Source: PLoS One. 2016 Dec 14;11(12):e0167960. doi: 10.1371/journal.pone.0167960 (PMC5156396; doi:10.1371/journal.pone.0167960)
Supplement: S1 Appendix — (DOCX) [file pone.0167960.s001.docx]

**S1 Appendix**

**Inverse Linear Quadratic Gaussian Model (LQG)**

We formulate the driving task as a LQG problem with a linear dynamic system and a quadratic reward function. In forward LQG problems, the optimal controller generates an optimal control policy that maximizes a given reward function. In inverse LQG problems, we use observed movements to infer the underlying reward function that best explains the observed behavior.

**Linear dynamic system** Assuming the driving task as a linear dynamic system with a partial hidden state *Xt*  (Equation 5) and observable feedback *Zt* (Equation 6)

With:

$A=\left[ \begin{matrix} a & b & 0 \\ 0 & －\beta& 0 \\ \gamma& 0 & -\gamma\end{matrix} \right]$ (8)

Β =$\left[ \begin{matrix} 0 \\ \beta\\ 0 \end{matrix} \right]$ (9)

C = [0,0,1] (10)

In which, *a*, *b* are car dynamics parameters (assuming known), *Vt* is Gaussian noise, β and γ are motor and perceptual speed that are estimated from Task 1. Note that in the state *Xt*, the hidden true car position and perceived car position are measured as a distance to goal stopping position (parameterized as the goal state in the reward function), which we will estimate through MLE using observed behavior.

**Quadratic reward function** **and goal state (*G)* and motivation (*M) parameters.*** We assume the reward function $r\left( X_{t}, U_{t} \right)$is a function that evaluates the state *Xt* based on its distance from the goal state *G* (through $g\left( X_{t}, G \right)$), and the action $U_{t}$ (through $U_{t}^{2}q$).

Reward function: $r\left( X_{t}, U_{t} \right)=g\left( X_{t}, G \right)-U_{t}^{2}q$ (11)

Without loss of generality, let q = 1 (i.e. optimal action will not change if scaling the reward function), thus *r*(*Xt*,*Ut*) is a function of goal state *G* and motivation *M,*  in which M is defined as the ratio of the distance between current state and the goal state over the energy expenditure. We assume subjects were using a stationary (infinite horizon) policy and the reward function has a diagonal form (i.e. no joint influence between state elements in the reward function).

In LQG setting, subjects first estimate true state from observation using a Kalman filter to convert the problem to a fully observable system, and then solve it as a LQR (Linear- Quadratic-Regulator) problem:

$d\hat{X_{t}}=A\hat{X_{t}}dt+BU_{t}dt+L_{t}\left( Z_{t}-C\hat{X_{t}} \right)dt$ (12)

$U_{t}= -\boldsymbol{k}\hat{X_{t}}$ (13)

In which $L_{t}$ is Kalman gain, $U_{t}$ is a linear combination of the states and ***k*** can be estimated from $U_{t}$ and recorded behavior data through linear regression. This suggests a quadratic value function:

$v\left( \hat{x},t \right)= -\frac{1}{2}\hat{x_{t}}'w\hat{x_{t}}$ (14)

$\nabla_{\hat{x}}v\left( \hat{x},t \right)= -w\hat{x_{t}}$ (15)

where w is an unknown symmetric matrix. Assuming a stationary policy and infinite horizon, the HJB equation will be:

$g\left( \hat{x} \right)= \hat{x}^{'}A'w\hat{x}+\frac{1}{2}u^{'}qu-u'B' \nabla_{\hat{x}}v\left( \hat{x},t \right)-\frac{1}{2}Tr(cc^{'}w)$ (16)

Since

$u_{t}^{*}=q^{-1}'B' \nabla_{\hat{x}}v\left( \hat{x},t \right)$ (17)

Without loss of generality, let q = 1, thus equation (16) can be simplified to:

$g\left( \hat{x} \right)= -\frac{1}{2}\hat{x}'(-2A^{'}w+\boldsymbol{k}^{'}\boldsymbol{k})\hat{x}$ (18)

In which we define *M* as motivation:

$g\left( \hat{x} \right)= -\frac{1}{2}\hat{x}'M\hat{x}$ (19)

$M=-2A^{'}w+\boldsymbol{k}'\boldsymbol{k}$ (20)

In which *A* and ***k*** are known from Equation 8 and Equation 13, and *w* can be solved by using optimal LQR solution based on Equation 17.

Reference:

Movellan J. R. (2011) Continuous Time Stochastic Optimal Control MPLab Tutorials, University of California San Diego
