# Supplementary material for: Using Optimal Control to Disambiguate the Effect of Depression on Sensorimotor, Motivational and Goal-Setting Functions
Source: PLoS One. 2016 Dec 14;11(12):e0167960. doi: 10.1371/journal.pone.0167960 (PMC5156396; doi:10.1371/journal.pone.0167960)
Supplement: S1 Fig — (DOCX) [file pone.0167960.s002.docx]

**S1 Figure**

**Figure A.** Influences of model parameters. Left (β): higher motor speed lead to faster arrival time to target; Middle (G): different goal distances lead to different stopping position; Right (M): higher motivational level lead to faster arrival time to the goal.

**Figure B.** Sensorimotor speed in depressive groups. Left: sensory speed. Right: motor speed. (* p<.05, ** p<.01)

**Figure C**. Goal stop distance in depressive groups. (* p<.05, ** p<.01, ** p<.001)

**Figure D.** Motivation in depressive groups over time.

**Figure E.** Action cost: model simulation and data. Left: Model Simulation of action cost as a function of Goal distance (G) and Motivation (M). G=0 is where the stop sign is at. Right: Model prediction of action cost based on estimated Goal distance and Motivation from behavioral data.

**Figure F.** Pairwise comparison among model parameters and BDI. Left: Pairwise mutual information. Numbers are pairwise mutual information among goal state (G, distance to stop-sign), motivation (M), sensorimotor speed (β) and BDI. Pairwise mutual information is a more generalized correlation measure, also captures non-linear relationship. Right: pairwise correlation coefficient. Numbers are correlation coefficient among goal state (G), motivation (M), sensorimotor speed (β) and BDI.
